# The Association between Hypertension and Insomnia: A Bidirectional Meta-Analysis of Prospective Cohort Studies

**DOI:** 10.1155/2022/4476905

**Published:** 2022-12-29

**Authors:** Dingwei Liu, Chao Yu, Ke Huang, Shawn Thomas, Wei Yang, Song Liu, Jie Kuang

**Affiliations:** ^1^Department of Gastroenterology, The First Affiliated Hospital of Nanchang University, Nanchang, China; ^2^Center for Prevention and Treatment of Cardiovascular Diseases, The Second Affiliated Hospital of Nanchang University, Nanchang, China; ^3^The First Clinical Medical College, Nanchang University, Nanchang, China; ^4^School of Public Health, University of Nevada, Reno, USA; ^5^Science and Technology Department, The Second Affiliated Hospital of Nanchang University, Nanchang, China; ^6^Jiangxi Provincial Key Laboratory of Preventive Medicine, School of Public Health, Nanchang University, Nanchang, China

## Abstract

**Background:**

Studies on bidirectional associations between hypertension and insomnia are inconclusive. The purpose of this meta-analysis was to systematically review and summarize the current evidence from epidemiological studies that evaluated this relationship.

**Materials and Methods:**

PubMed, Embase, China National Knowledge Infrastructure (CNKI), Wan Fang, and VIP databases were searched for studies published up to May 2021. Prospective cohort studies that reported the relationship between hypertension and insomnia in adults were included. Data were extracted or provided by the authors according to the prevalence rate, incidence rate, unadjusted or adjusted odds ratio (OR), and 95% confidence interval (CI). Heterogeneity was assessed by I2 statistics. ORs were pooled by using random-effects models.

**Results:**

A total of 23 prospective studies were identified. Twenty cohort studies recorded OR-adjusted value with the outcome for hypertension (OR = 1.11, 95% CI: 1.07–1.16; I2 = 83.9%), and three cohort studies reported OR-adjusted value with the outcome for insomnia (OR = 1.20, 95%CI: 1.08–1.32; I2 = 35.1%). Subgroup analysis showed that early morning awakening and composite insomnia were significantly associated with hypertension.

**Conclusions:**

The result indicates a possible bidirectional association between hypertension and insomnia. Early identification and prevention of insomnia in hypertension patients are needed, and vice versa.

## 1. Introduction

Hypertension affects 26.4% of people worldwide and is considered the main risk factor for mortality [1]. Patients with hypertension commonly complain of insomnia. Hypertension adults have reported an increased risk of insomnia, with a risk ratio of 1.5 to 3.18 [2, 3]. Several studies have reported that adults with hypertension have an increased risk of insomnia. Still, patients with hypertension also suffer from psychological diseases such as anxiety and depression [4–6], which are risk factors for insomnia. However, there is no systemic evidence available to support this relationship.

Insomnia is the most common sleep disorder and the second most prevalent mental disorder worldwide. It is defined as the occurrence of difficulty initiating sleep (DIS) or difficulty falling asleep (DFA), sleep continuity disturbance (SCD) or difficulty maintaining asleep (DMS), non-restorative sleep (NRS), and early morning awakening (EMA) [7–9]. About 17%–19% of the US population presented insomnia symptoms [10]. Nearly 15% of the population in China reported insomnia [11].

Insomnia is associated with a variety of mental and physical health problems. In addition, abnormal sleepers may also suffer from cardiovascular disease [12]. Hernandez-Aceituno et al. found that increased use of antihypertensive medications was significantly associated with poor sleep status [13]. Therefore, treating insomnia and ameliorating sleep habits may be crucial to control some chronic diseases [8].

Hypertension and insomnia are major public health issues, and investigations into the association between these diseases have recently attracted broad attention [14]. Li et al. performed a meta-analysis to assess the pooled relative risk (RR) of insomnia on hypertension. The findings suggested that the ultimate RR value was 1.21 (1.10 to 1.33) [15]. However, in various epidemiological studies, this association remains inconsistent [16–19], and comprehensive reviews that focus on the bidirectional association between insomnia and hypertension are lacking [20]. Therefore, we conducted a bidirectional systemic review and meta-analysis to determine the association between insomnia and hypertension.

## 2. Materials and Methods

This meta-analysis was performed according to the Preferred Reporting Items for Systematic Reviews and Meta-Analyses (PRISMA) guidelines. Two authors (HK and LDW) independently evaluated eligibility, extracted data, and scored the quality of the study included. Disagreements were settled by a discussion until consensus was reached or determined by a third author (KJ).

### 2.1. Search Strategy

We searched PubMed, Embase, CNKI, Wan Fang, and VIP (up to May 2021). To minimize bias, two authors (HK and LDW) independently performed an online search using the following combination of search terms: “hypertension,” “high blood pressure,” “disorders of initiating and maintaining sleep,” “sleep disturbance,” “sleep disorder,” “sleep quality,” “insomnia,” “agrypnia,” and “sleep maintenance,” to identify published studies evaluating the association between hypertension and insomnia. Additionally, a search of the reference lists of eligible articles was conducted to determine any missed reports.

### 2.2. Inclusion and Exclusion Criteria

Studies were selected based on the following inclusion criteria: (1) the study design was prospective; (2) participants aged 18 years or older; (3) insomnia diagnosed through any symptoms (DIS, DFA, SCD, NRS, DMS, and EMA) or diagnostic criteria (e.g., DSM-IV/V, ICSD-1/2/3, and ICD-9/10); (4) hypertension diagnosed was based on a current resting systolic blood pressure (SBP) ≥ 140 mmHg and/or diastolic blood pressure (DBP) ≥ 90 mmHg, by self-reported hypertension, or by antihypertensive treatment; (5) included an OR value and a 95% confidence interval or other sufficient results; (6) published in English and Chinese. The exclusion criteria included the following: (1) studies with special populations (e.g., child and pregnancy); (2) letters, comments, reviews, or meta-analyses; and (3) the full text was not available.

### 2.3. Risk of Bias Assessment

The methodological quality for the included studies was assessed based on the Newcastle-Ottawa Scale (NOS), including the quality of study selection (0–4 points), comparability (0–2 points), and exposure and outcome of study participants. A final score ≥7 is considered a high-quality article [21].

### 2.4. Data Extraction

Two researchers (HK and LDW) independently extracted the following information from each study: basic information (author, publication time, nationality, source of literature, number of studies, age, and gender), criteria for evaluation of hypertension and insomnia, number of patients and participants, crude, adjusted OR and confidence intervals, and the variables used in multivariate analyses. A maximum level adjustment was selected if adjusted ORs were shown in different adjustment levels.

### 2.5. Statistical Analysis

The association between hypertension and insomnia was assessed from the following perspectives: (1) the OR of baseline insomnia and risk of incident hypertension in prospective cohort studies; (2) the OR of baseline hypertension and risk of future insomnia in prospective cohort studies. If the studies reported effect size other than OR, the transformation was performed, and unpublished data were collected by contacting the corresponding author if possible. A random-effects model was used to pool the data, and statistical heterogeneity between summary data was evaluated using the I2 statistic. 25%, 50%, and 75% represent low, moderate, and high heterogeneity [22]. Forest plots were used for the graphical display of the results. Funnel plot, Begg's test, and Egger's test were used to assess publication bias. Visual asymmetry in funnel plot or *P* ≤ 0.05 in Begg's and Egger's tests was considered statistically significant. Subgroup analyses were performed to illustrate the influence of study results' specific characteristics, including age, sex, insomnia type, continent, hypertension assessment, insomnia assessment, and follow-up time. Based on hypertension assessment, studies were divided into different types: SBP ≥ 140 mmHg and/or DBP ≥ 90 mmHg or use of antihypertensive medication and others (self-report or different levels of BP or ICD9/10). For insomnia assessment, studies using sleep questionnaires were defined as “non-clinical insomnia criteria,” and others such as DSM-IV and ICSD-I were defined as “clinical diagnostic criteria.” Sensitivity analyses were conducted to detect the stability of our results by excluding each included study one at a time. Stata version 16.0 (StataCorp, College Station, TX) was used for all statistical analyses. *P* values were two-sided, and a significance level cutoff of 0.05 was used.

## 3. Results

### 3.1. Study Selection and Characteristics

**Figure 1:**
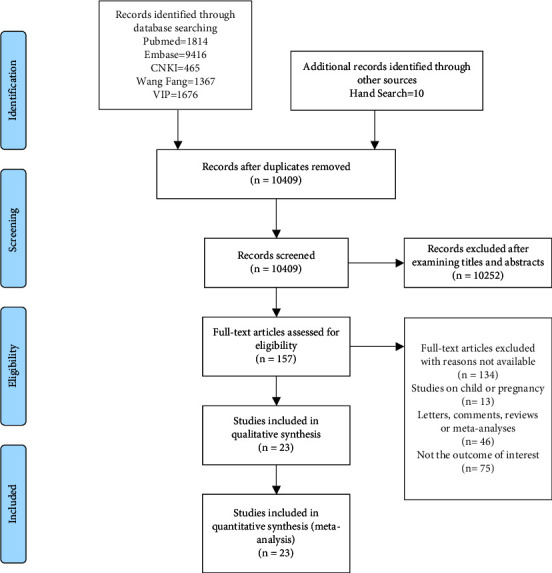
Flowchart of the article selection process.

The literature search yielded a total of 14,738 articles. After removing the duplicate articles, 10,409 articles remained. After reviewing the titles and abstracts, articles were excluded for their irrelevance. The remaining 157 articles were identified through full-text screening. One hundred thirty-four articles did not meet the inclusion criteria, and 23 were included. After quality assessment, most studies have shown good quality with scores ranging from 6 to 8.95. 65% (22/23) of the studies' score was ≥6, and 43.48% (10/23) of the studies' score was ≥8. However, three studies did not satisfy the criterion “Demonstration that outcome of interest was not present at start of study.” Two studies [23, 24] did not meet the “Demonstration that outcome of interest was not present at the start of the study” criterion in insomnia predicting incident hypertension. One study [25] did not meet the criterion in hypertension predicting incident insomnia (Supplementary Table 1). The final meta-analysis (Figure 1) included 23 cohort studies (Table 1).

### 3.2. Cohort Studies of Baseline Insomnia Predicting the Risk of Hypertension

The association between samples with insomnia at baseline and incident hypertension was investigated in twenty studies, with a total of 242,415 participants. Table 1 summarizes the basic characteristics of these studies. Of the 20 studies, hypertension was identified by measured blood pressure, self-reported hypertension, or antihypertensive treatment. Sleep questionnaire was used in four studies for diagnosing insomnia; one study used the Women's Health Initiative Insomnia Rating Scale (WHIIRS), and four studies used DSM-IV, ICSD-1, and ICD-9/10 instead. Seventeen studies were conducted in North America or Europe and three in Asia. The follow-up ranges from 1 to 20 years.

**Figure 2:**
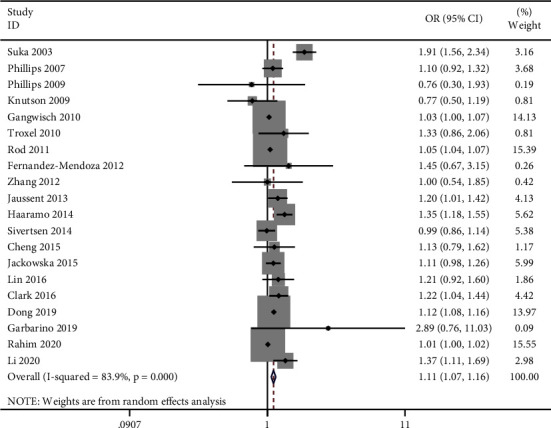
Forest plots of insomnia predicting incident hypertension. Squares represent the study-specific relative risk. Diamonds represent the summary relative risks (SRRs). Horizontal lines represent 95% confidence intervals (CIs).

The result was OR = 1.11 (95% CI: 1.07–1.16) with high heterogeneity (*I*^*2*^ = 83.9%, *P*  < 0.001) detected (Figure 2). Publication bias was found in the funnel plot (Supplementary Figure 1(a)) and confirmed by Egger's test (*P* = 0.01) but not in Begg's test (*P* = 0.347). We further performed subgroup analyses (Table 2). The association between insomnia and hypertension were significant in the age subgroups (40−60 vs. < 40 y: OR=1.10, 95% CI: 1.05−1.15, *I*^*2*^ = 85.2%, *P* < 0.001; >60 vs. < 40 y: OR=1.12, 95% CI: 1.09−1.16, *I*^*2*^ = 0%, *P* < 0.810). In male (the proportion of males in each study <40%) (OR = 1.10, 95%CI: 1.03–1.17, *I*^*2*^ = 81.0%, *P* < 0.001) and male (the proportion of males in each study: 40%–60%) (OR = 1.12, 95%CI: 1.08–1.15, *I*^*2*^ = 15.4%, *P* = 0.294) groups, we also found an association between insomnia and incident hypertension. Studies in North America (OR = 1.07, 95%CI: 1.01–1.13, *I*^*2*^ = 79.1%, *P* < 0.001) and Europe (OR = 1.14, 95%CI: 1.04–1.25, *I*^*2*^ = 72.5%, *P* = 0.001) found that insomnia was significantly associated with increased risk of hypertension, while studies in Asia did not. However, in Asia, the estimate was based on only three studies. More studies are needed to get more accurate values. The subgroup that included the insomnia subtype found that early morning awakening (EMA) (OR = 1.13, 95%CI: 1.07–1.20, *I*^*2*^ = 0%, *P* = 0.566) and composite insomnia (OR = 1.12, 95%CI: 1.06–1.17, *I*^*2*^ = 79.9%, *P* < 0.001) were associated with incident hypertension. In the subgroup of insomnia assessment, non-clinical insomnia criteria (OR = 1.12, 95%CI: 1.07–1.17, *I*^*2*^ = 86.3%, *P* < 0.001) showed an association between insomnia and incident hypertension. As for hypertension assessment, significant associations were found both in SBP ≥ 140 mmHg and/or DBP ≥ 90 mmHg or use of the antihypertensive medication group (OR = 1.21, 95%CI: 1.10–1.33, *I*^*2*^ = 86.9%,*P* < 0.001) and other (self-report or different levels of BP or ICD9/10) group (OR = 1.04, 95%CI: 1.01–1.08, *I*^*2*^ = 66.3%, *P* = 0.001). Sensitivity analyses were then performed, and the results showed that the estimated ORs were still statistically significant (Supplementary Figure 2(a)). We also drew a forest plot after excluding two studies [23, 24] that did not satisfy the “Demonstration that outcome of interest was not present at the start of the study” criterion (Supplementary Figure 3(a)). The figure shows that excluding the two studies does not influence the main results. We have also plotted the effect size against follow-up time to confirm there is no link between the two (Supplementary Figure 4(a)).

### 3.3. Cohort Studies of Baseline Hypertension Predicting the Risk of Insomnia

**Figure 3:**
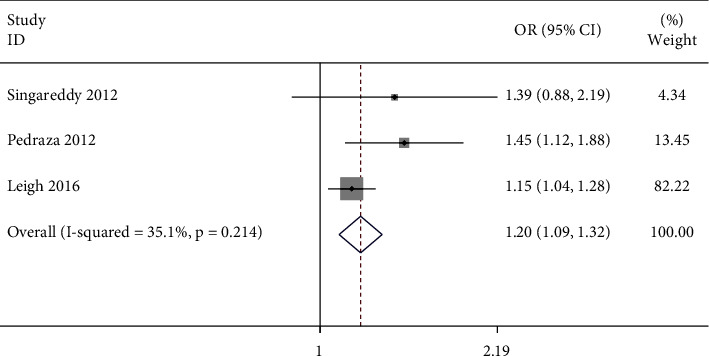
Forest plots of hypertension predicting incident insomnia. Squares represent the study-specific relative risk. Diamonds represent the summary relative risks (SRRs). Horizontal lines represent 95% confidence intervals (CIs).

The samples with hypertension at baseline and incident insomnia were investigated in three studies, including 13,052 participants, as shown in Table 1. Of the three studies, hypertension was identified by SBP and (or) DBP ≥ 140/90, self-reported hypertension, or antihypertensive treatment. Sleep questionnaires were used in three studies for diagnosing insomnia. Two studies were conducted in the USA and one in Australia. The follow-up ranges from 3 to 15 years. Three reported ORs were included in the pooled result. The result was OR = 1.20 (95% CI: 1.08–1.32) with low heterogeneity detected (*I*^*2*^ = 35.1%, *P* = 0.214) (Figure 3). However, the pooled OR and 95% CI were based on only three studies, which will affect the results. We drew a funnel plot (Supplementary Figure 1(b)) to show the general condition of each study and performed Begg's and Egger's tests to identify the publication bias. No publication bias was found by Begg's (*P* = 1.000) and Egger's test (*P* = 0.332). Sensitivity analyses showed that the estimated ORs were still statistically significant (Supplementary Figure S2(b)). We also drew a forest plot after excluding one study [25] that did not satisfy the “Demonstration that outcome of interest was not present at the start of the study” criterion (Supplementary Figure S3(b)). The figure shows that excluding the study does not influence the main results. We have also plotted the effect size against follow-up time to confirm there is no link between the two (Supplementary Figure 4(b)).

## 4. Discussion

It is the first meta-analysis to investigate the bidirectional association between insomnia and hypertension as far as we know. This meta-analysis indicated a likely bidirectional association between insomnia and hypertension in the prospective cohort studies.

Our results suggested that insomnia and hypertension are significantly related. We collected adjusted ORs to test their association, and we found that the OR of insomnia predicting hypertension was 1.11 (95%CI: 1.07–1.16), and the OR of hypertension predicting insomnia risk was 1.20 (95% CI: 1.08–1.32). When stratified by insomnia assessment, we found an association only between insomnia diagnosed by non-clinical criteria and hypertension, not with insomnia diagnosed by clinical criteria. Studies that used non-clinical criteria, such as sleep questionnaires, may have a higher sensitivity to detect missed sleep issues when collecting only doctor-reported medical diagnoses. In addition, composite insomnia and early morning awakening were found to be significantly associated with hypertension, consistent with the previous meta-analysis [42]. It is also worth noting that we found a more substantial effect in using SBP ≥ 140 mmHg and/or DBP ≥ 90 mmHg or antihypertensive medication group (OR = 1.21, 95%CI: 1.10–1.33) compared with the other group (self-report or different levels of BP or ICD9/10) (OR = 1.04, 95%CI: 1.01–1.08). The reason may be that some patients tended not to report hypertension without knowing the diagnostic criteria, so patients with hypertension can be classified as not hypertensive [43]. Using different levels of BP may decrease the number of people diagnosed with hypertension. As for gender difference, men with insomnia were more likely to suffer from hypertension than women [38].

Although our study confirmed that insomnia was associated with an increase in hypertension occurrence, the mechanism behind it was not fully elucidated. Generally, insomnia affects blood pressure through 3 pathways. (1) Psychogenic pathways: insomnia leads to mental changes, mainly manifested as anxiety [42], depression [38], and so on. The sympathetic nervous system becomes overactive leading to peripheral vasoconstriction and blood pressure increase. (2) Neurogenic pathway: it was found that the activity of the sympathetic nervous system (SNS) increased in insomnia patients, which would lead to a series of hypertension events [44, 45]. (3) Humoral pathway: insomnia has been proven to increase the release of pulsatile cortisol by affecting its rhythm [46]. In addition, insomnia causes stress dysregulation [47], which is a potential cause of high hypothalamic-pituitary-adrenal (HPA) reactivity [48]. The renin-angiotensin-aldosterone system (RAAS) was also activated along with the HPA axis [49]. In addition, insomnia is a pathological state accompanied by inflammation, oxidative stress, and endothelial dysfunction [47], which may be the potential mechanism of hypertension [50]. At the same time, the melatonin secretion of patients with hypertension could be disturbed [47], circadian rhythm would change, and sleep disorders would occur [8, 47]. Indeed, these mechanisms may interact with subsequent pathological conditions. Besides, nocturnal hypertension may also be a likely link between insomnia and increased blood pressure, which may be triggered by specific triggers (OSA episode, arousal, rapid-eye-movement sleep, and nocturia). However, few studies investigate the biological plausibility between hypertension and insomnia. The exact mechanism between hypertension and insomnia still needs further elucidation.

This meta-analysis has several strengths and limitations to address. The primary strength is that this is the first meta-analysis that comprehensively examines the bidirectional association of insomnia and hypertension based on a comprehensive literature search of studies. Our meta-analysis provided more reliable results in cohort studies with a larger number of studies than the previous meta-analysis. In addition, our study quantifies the bidirectional association in detail, which was stratified according to factors such as gender, continents, hypertension assessment, and different types of insomnia. Insomnia assessment based on formal criteria was first considered in the current studies, which helps to shed new light on the exact effect of different criteria. However, there are still some limitations in this study. First, the heterogeneity is high in some analyses of insomnia and hypertension. By subgroup analysis, different types of hypertension assessment and insomnia subtypes might be the source of the heterogeneity. Secondly, some studies failed to control potential confounders such as psychological symptoms, age, or gender, although most studies adjusted several factors. Thirdly, publication bias of baseline insomnia and risk of hypertension was found in the funnel plot and identified by Egger's test (*P*=0.01). Three studies have different hypertension assessments than most included studies, which may be why they are outside the confidence interval of the funnel plot. Fourth, our analyses did not consider the effect of sleep time on hypertension because insomnia patients often have abnormal sleep time, which could bias our research results. Finally, the results of baseline hypertension predicting the risk of insomnia were based on only three studies, which will affect the stability of the meta-analysis. So, there is an increasing demand for high-quality research in the future.

## 5. Conclusions

In summary, our study shows that there may be a statistically significant bidirectional association between hypertension and insomnia. Early morning awakening and composite insomnia are potential risk factors for hypertension, while baseline hypertension also serves as a risk factor for insomnia. An assessment of insomnia may be beneficial for patients with hypertension, and treatment for hypertension may include improving sleep quality in those patients who show significant clinical symptoms of insomnia.

## Figures and Tables

**Table 1:** Characteristics of cohort studies in the meta-analysis.

Author	Year	Country	Follow-upyears	Sample	Age range (or mean age)	Insomnia assessment	Insomnia type	Hypertension assessment	Note
Suka et al. [26]	2003	Japan	4	9237	40–55 y	Sleep questionnaire	DIS, DMS	(1) SBP ≥ 140 mmHg and (or) DBP ≥ 90 mmHg	Baseline insomnia
								(2) Antihypertensive treatment	NOS rating: 6
Phillips and Mannino [27]	2007	USA	6	8,757	45–69 y	Sleep questionnaire	DFA, SCD, NRS	(1) SBP ≥ 160 mmHg and (or) DBP ≥ 95 mmHg	Baseline insomnia
								(2) Antihypertensive treatment	NOS rating: 8
Phillips et al. [28]	2009	USA	6	1,419	64–91 y	Sleep questionnaire	D4FA, EMA, SCD	(1) SBP ≥ 140 mmHg and (or) DBP ≥ 90 mmHg	Baseline insomnia
								(2) Antihypertensive treatment (3) Self-reported hypertension	NOS rating: 7
Knutson et al. [29]	2009	USA	5	535	35–45 y	Sleep questionnaire	DMS	(1) SBP≥140 mmHg and (or) DBP ≥ 90 mmHg	Baseline insomnia
								(2) Antihypertensive treatment	NOS rating: 6
Gangwisch et al. [30]	2010	USA	10	4,913	32–86 y	Sleep questionnaire	DIS, DMS, EMA	(1) SBP≥140 mmHg and (or) DBP ≥ 90 mmHg	Baseline insomnia
								(2) Self-reported hypertension (3) Physician or hospital diagnosis	NOS rating: 7
Troxel et al. [31]	2010	USA	3	812	45–74 y	Sleep questionnaire	DIS, NRS	(1) SBP ≥ 130 mmHg and (or) DBP ≥ 85 mmHg	Baseline insomnia
								(2) Antihypertensive treatment	NOS rating: 6
Rod et al. [23]	2011	France	20	16,989	36–52 y	Sleep questionnaire	DFA, DMS, EMA	(1) Self-reported hypertension	Baseline insomnia
									NOS rating: 6
Fernandez-Mendoza et al. [30]	2012	USA	7.5	786	*M* = 47.5 y	Sleep questionnaire	DFA, DMS, EMA, NRS, chronic insomnia	(1) SBP ≥ 140 mmHg and (or) DBP ≥ 90 mmHg	Baseline insomnia
								(2) Self-reported hypertension (3) Antihypertensive treatment	NOS rating: 7
Pedraza et al. [31]	2012	USA	3	1,085	≥75 y	Sleep questionnaire	DIS, DMS, NRS	(1) SBP ≥ 140 mmHg and (or) DBP ≥ 90 mmHg	Baseline HTN
								(2) Self-reported hypertension (3) Physician or hospital diagnosis	NOS rating: 6
Singareddy et al. [32]	2012	USA	7.5	1,246	≥20 y	Sleep questionnaire	DFA, DMS, EMA, NRS	(1) Antihypertensive treatment	Baseline HTN
								(2) Self-reported hypertension	NOS rating: 6
Zhang et al. [19]	2012	China	5.2	2,316	*M* = 47.5 y	DSM-IV, ICSD-1, ICD-10	DIS, DMS, EMA	(1) Self-reported hypertension	Baseline insomnia
									NOS rating: 8
Jaussent et al. [33]	2013	France	6	5,494	65–94 y	Sleep questionnaire	Eds, DIS, DMS, EMA	(1) SBP≥160 mmHg and (or) DBP ≥ 95 mmHg	Baseline insomnia
								(2) Antihypertensive treatment	NOS rating: 6
Haaramo et al. [34]	2014	Finland	5	6,477	40–60 y	Sleep questionnaire	DFA, DMS	(1) Antihypertensive treatment	Baseline insomnia
									NOS rating: 7
Sivertsen et al. [35]	2014	Norway	11	24,715	20–89 y	DSM-IV	DMS, DIS, NRS	(1) Self-reported hypertension	Baseline insomnia
									NOS rating: 8
Jackowska and Steptoe [36]	2015	UK	4	3,937	≥50 y	Sleep questionnaire	DFA, DMS, EMA	(1) SBP ≥ 140 mmHg and (or) DBP ≥ 90 mmHg	Baseline insomnia
								(2) Antihypertensive treatment	NOS rating: 6
Cheng et al. [12]	2015	USA	1	967	*M* = 43.1 y	Sleep questionnaire	DFA, DMS	(1) Self-reported hypertension	Baseline insomnia
									NOS rating: 6
Clark et al. [37]	2016	Finland	1	70,049	18–69 y	DSM-IV	DFA, DMS, EMA, NRS	(1) Antihypertensive treatment	Baseline insomnia
									NOS rating: 8
Leigh et al. [38]	2016	Australia	15	10,721	70–75 y	Sleep questionnaire	EMA, DIS	(1) Self-reported hypertension	Baseline HTN
									NOS rating: 6
Lin et al. [24]	2016	Taiwan	5	44,559	>20 y	1.ICD-9	DFA, DMS, EMA	(1) ICD-9	Baseline insomnia
									NOS rating: 7
Dong and Yang [38]	2019	USA	8	18,123	≥50 y	Sleep questionnaire	DFA, DMS, EMA, NRS	(1) SBP ≥ 140 mmHg and (or) DBP ≥ 90 mmHg	Baseline insomnia
								(2) Antihypertensive treatment	NOS rating: 6
Garbarino and Magnavita [39]	2019	Italy	5	234	*M* = 36 y	Sleep questionnaire	DIS, EMA, NRS	(1) SBP ≥ 130 mmHg and (or) DBP ≥ 85 mmHg	Baseline insomnia NOS rating: 5
Rahim et al. [40]	2020	Canada	12	2,079	35–69 y	Sleep questionnaire	DIS, DMS, NRS	(1) ICD-9, ICD-10	Baseline insomnia NOS rating: 6
Li et al. [41]	2020	USA	6	6,965	18–74 y	Women's Health Initiative Insomnia Rating Scale (WHIIRS)	NA	(1) SBP ≥ 140 mmHg and (or) DBP ≥ 90 mmHg	Baseline insomnia NOS rating: 7
								(2) Antihypertensive treatment	

DBP = diastolic blood pressure; DFA, difficulty falling asleep; DIS, difficulty initiating sleep; DMS, difficulty maintaining sleep; DSM, Diagnostic and Statistical Manual of Mental Disorders; EMA, early morning awakening; HTN = hypertension; ICSD, International Classification of Sleep Disorders; ICD, International Classification of Diseases; NA = not available; NOS = Newcastle-Ottawa Scale; NRS, non-restorative sleep; SBP = systolic blood pressure.

**Table 2:** Subgroup analyses of the association between insomnia and hypertension.

	No. of reports	OR (95% CI)	*P*value for heterogeneity	*I* ^2^ (%)
Subgroup analyses				
Age				
<40	1	2.89 (0.76–11.07)	—	—
40–60	14	1.10 (1.05–1.15)	<0.001	85.2
>60	5	1.12 (1.09–1.16)	0.810	0
Sex^1^				
Male (<40%)	6	1.10 (1.03–1.17)	<0.001	81.0
Male (40%–60%)	12	1.12 (1.08–1.15)	0.294	15.4
Male (>60%)	2	1.40 (0.78–2.52)	<0.001	97
Continent				
North America	10	1.07 (1.01–1.13)	<0.001	79.1
Asia	3	1.40 (0.94–2.08)	0.011	77.9
Europe	7	1.14 (1.04–1.25)	0.001	72.5
Follow-up time				
>5	11	1.06 (1.02–1.10)	<0.001	81.9
≤5	9	1.27 (1.12–1.43)	0.008	55.6
Insomnia subtype^2^				
DIS/DFA	4	1.26 (0.87–1.84)	0.004	77.9
DMS	4	1.17 (0.89–1.55)	0.001	83.0
EMA	2	1.13 (1.07–1.20)	0.566	0
NRS	1	1.39 (0.78–2.48)	—	—
Composite insomnia	15	1.12 (1.06–1.17)	<0.001	79.9
Hypertension assessment				
SBP ≥ 140 mmHg and/or DBP ≥ 90 mmHg or use of antihypertensive medication	9	1.21 (1.10–1.33)	<0.001	86.9
Others (self-report or different levels of BP or ICD9/10)	11	1.04 (1.01–1.08)	0.001	66.3
Insomnia assessment				
Clinical diagnostic criteria	3	1.03 (0.91–1.17)	0.448	0
Non-clinical insomnia criteria	17	1.12 (1.07–1.17)	<0.001	86.3

CI, confidence interval; OR, odds ratio. HTN, hypertension; BP, blood pressure; EMA: early morning awakening; DMS, difficulty maintaining sleep; DIS; difficulty initiating sleep; DFA; difficulty falling asleep; NRS; non-restorative sleep. ^1^The variable “sex” was used as a continuous variable (according to the proportion of males in each study). ^2^One study provided data on DMS and DIS/DFA. Two studies provided data on DMS, DIS/DFA, and EMA. One study provided data on DIS/DFA and NRS. One study provided data only on DMS. Therefore, there are 26 reports from 20 studies.

## Data Availability

The data used to support the findings of this study are available from the corresponding author upon reasonable request.

## Supplementary Materials

Supplementary Materials Supplementary Table 1: quality assessment of included studies. Supplementary Figure 1: funnel plot with pseudo-95% confidence limits. Supplementary Figure 2: sensitivity analysis of the relationship between insomnia and hypertension. Supplementary Figure 3: funnel plot after excluding studies. Supplementary Figure 4: the correlation between follow-up time and effect size.
