# Supplementary material for: The Association between Hypertension and Insomnia: A Bidirectional Meta-Analysis of Prospective Cohort Studies
Source: Int J Hypertens. 2022 Dec 29;2022:4476905. doi: 10.1155/2022/4476905 (PMC9815923; doi:10.1155/2022/4476905)
Supplement: Supplementary Materials — Supplementary Table 1: quality assessment of included studies. Supplementary Figure 1: funnel plot with pseudo-95% confidence limits. Supplementary Figure 2: sensitivity analysis of the relationship between insomnia and hypertension. Supplementary Figure 3: funnel plot after excluding studies. Supplementary Figure 4: the correlation between follow-up time and effect size. [file 4476905.f1.zip › Supplementary Figures.pdf]

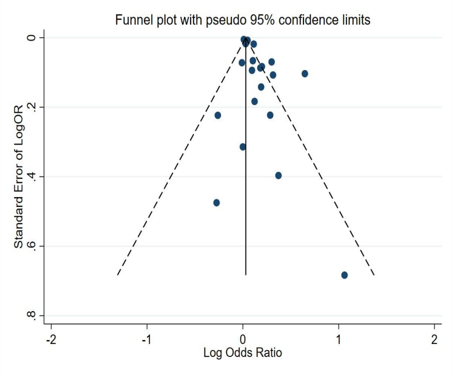

a

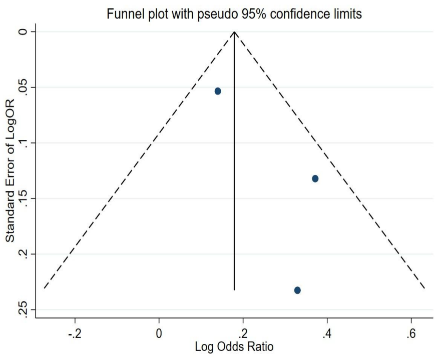

b

Supplementary Figure.1 Funnel plot with pseudo 95% confidence limits
(a) Cohort studies of insomnia predicting incident hypertension. (b) Cohort studies of hypertension predicting incident insomnia

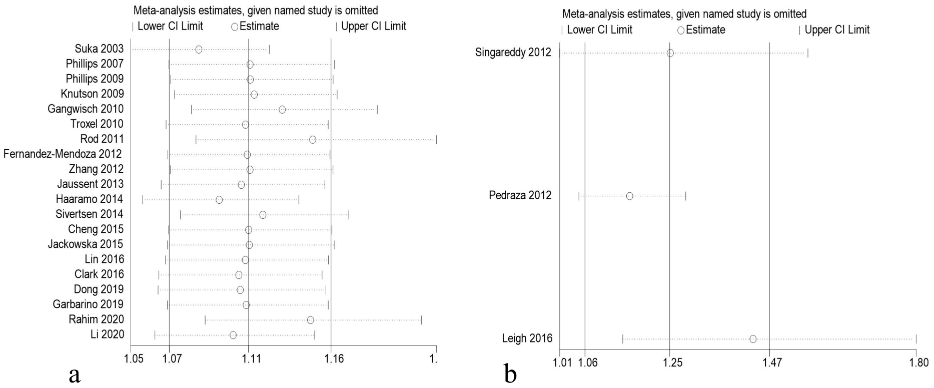

Supplementary Figure.2 Sensitivity analysis of the relationship between insomnia and hypertension (a) Cohort studies of insomnia predicting incident hypertension. (b) Cohort studies of hypertension predicting incident insomnia.

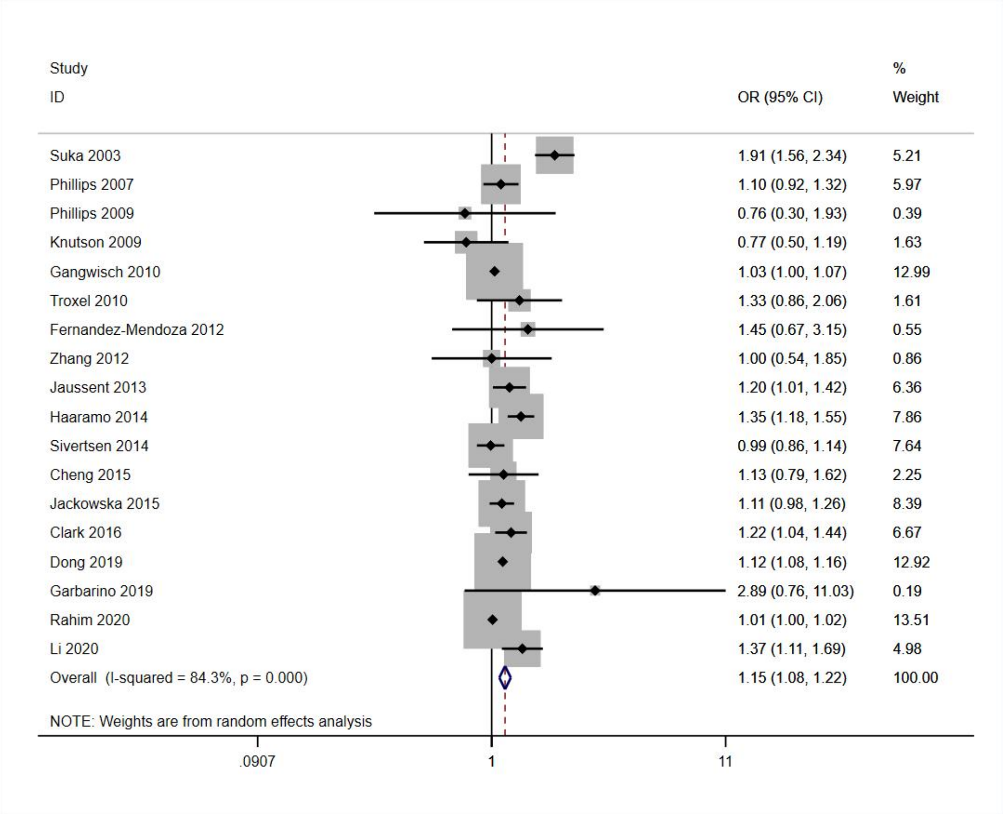

a

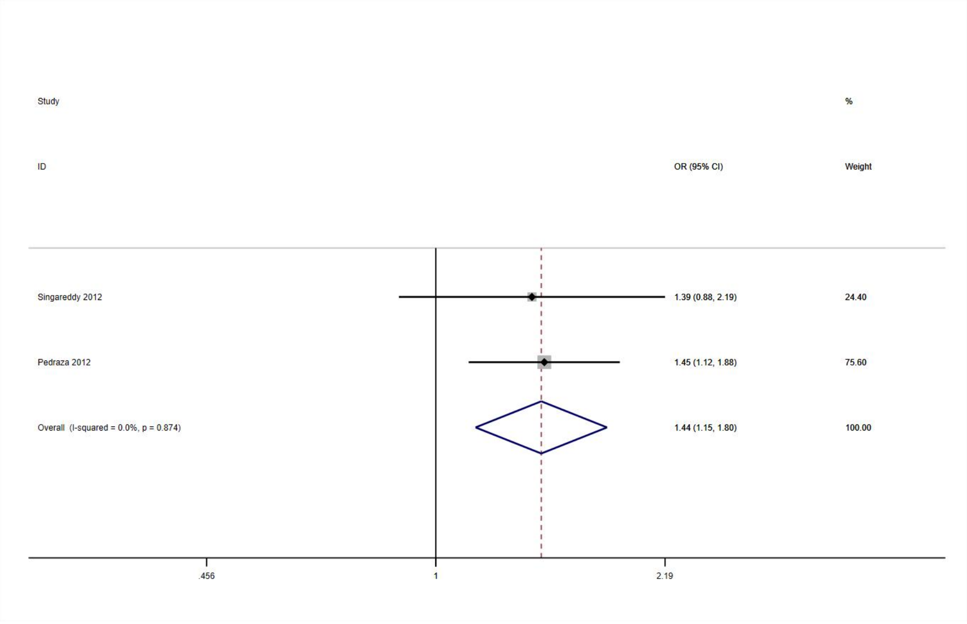

b

Supplementary Figure.3 (a) Forest plots of insomnia predicting incident hypertension after excluding two studies (Rod 2011, Lin 2016). (b) Forest plots of hypertension predicting incident insomnia after excluding one study (Leigh 2016).

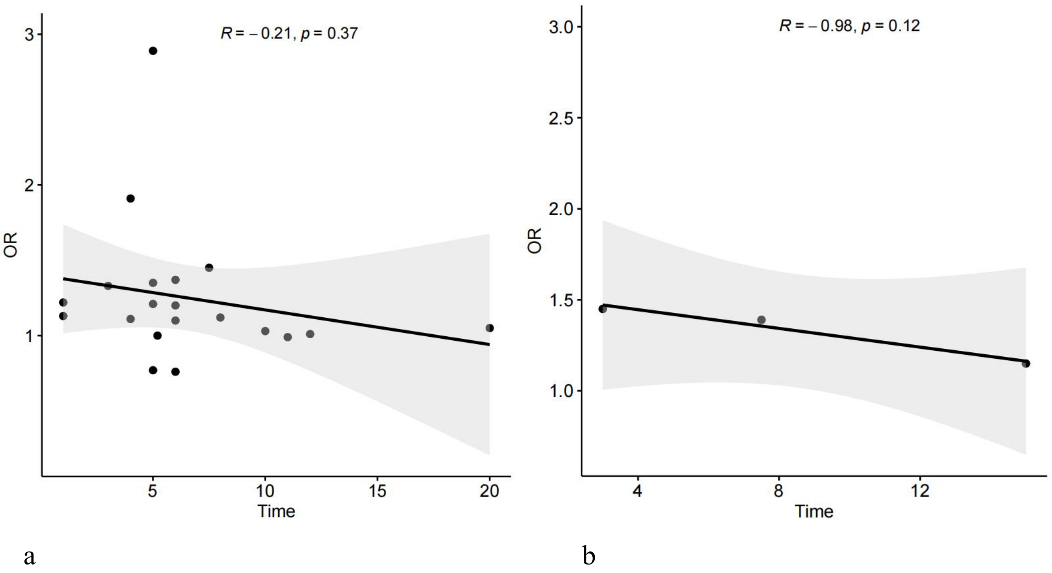

Supplementary Figure.4 (a) The correlation between follow-up time and effect size of insomnia predicting incident hypertension. (b) The correlation between follow-up time and effect size of hypertension predicting incident insomnia.
