# Supplementary material for: The Association between Hypertension and Insomnia: A Bidirectional Meta-Analysis of Prospective Cohort Studies
Source: Int J Hypertens. 2022 Dec 29;2022:4476905. doi: 10.1155/2022/4476905 (PMC9815923; doi:10.1155/2022/4476905)
Supplement: Supplementary Materials — Supplementary Table 1: quality assessment of included studies. Supplementary Figure 1: funnel plot with pseudo-95% confidence limits. Supplementary Figure 2: sensitivity analysis of the relationship between insomnia and hypertension. Supplementary Figure 3: funnel plot after excluding studies. Supplementary Figure 4: the correlation between follow-up time and effect size. [file 4476905.f1.zip › Supplementary Table1.docx]

| **Supplementary Table1** Quality assessment of included studies | | | | | | | | | | | |
| --- | --- | --- | --- | --- | --- | --- | --- | --- | --- | --- | --- |
| **Original studies** | | **Selection** | | | | **Comparability** | | | **Exposure** | | **Total score** |
|  |  | Representativeness of the exposed cohort | Selection of the non-exposed cohort | | Ascertainment of exposure | Demonstration that outcome of interest was not present at start of study | Comparability of cohorts on the basis of the design or analysis | Assessment of outcome | Was follow-up long enough for outcomes to occur | Adequacy of follow up of cohorts |  |
| Suka 2003 | | 0 | 1 | | 1 | 1 | 1 | 1 | 1 | 0 | 6 |
| Phillips 2007 | | 1 | 1 | | 1 | 1 | 1 | 1 | 1 | 1 | 8 |
| Phillips 2009 | | 1 | 1 | | 1 | 1 | 1 | 1 | 1 | 0 | 7 |
| Knutson 2009 | | 0 | 1 | | 1 | 1 | 1 | 1 | 1 | 0 | 6 |
| Gangwisch 2010 | | 1 | 1 | | 1 | 1 | 1 | 0 | 1 | 1 | 7 |
| Troxel 2010 | | 0 | 1 | | 1 | 1 | 1 | 0 | 1 | 1 | 6 |
| Rod 2011 | | 0 | 1 | | 1 | 0 | 2 | 0 | 1 | 1 | 6 |
| Fernandez-Mendoza 2012 | | 1 | 1 | | 1 | 1 | 2 | 0 | 1 | 0 | 7 |
| Pedraza 2012 | | 0 | 1 | | 1 | 1 | 1 | 1 | 1 | 0 | 6 |
| Singareddy 2012 | | 0 | 1 | | 1 | 1 | 1 | 0 | 1 | 1 | 6 |
| Zhang 2012 | | 1 | 1 | | 1 | 1 | 1 | 1 | 1 | 1 | 8 |
| Jaussent 2013 | | 1 | 1 | | 1 | 1 | 1 | 0 | 1 | 0 | 6 |
| Haaramo 2014 | | 1 | 1 | | 1 | 1 | 1 | 0 | 1 | 1 | 7 |
| Sivertsen 2014 | | 1 | 1 | | 1 | 1 | 2 | 0 | 1 | 1 | 8 |
| Jackowska 2015 | | 1 | 1 | | 1 | 1 | 1 | 1 | 0 | 0 | 6 |
| Cheng 2015 | | 1 | 1 | | 1 | 1 | 2 | 0 | 0 | 0 | 6 |
| Clark 2016 | | 0 | 1 | | 1 | 1 | 2 | 1 | 1 | 1 | 8 |
| Leigh 2016 | 1 | | | 1 | 1 | 0 | 1 | 1 | 1 | 0 | 6 |
| Lin 2016 | 1 | | | 1 | 1 | 0 | 2 | 1 | 1 | 0 | 7 |
| Dong 2019 | 1 | | | 1 | 1 | 1 | 1 | 0 | 1 | 0 | 6 |
| Garbarino 2019 | 1 | | | 1 | 1 | 1 | 1 | 0 | 0 | 0 | 5 |
| Rahim 2020 | 0 | | | 1 | 1 | 1 | 1 | 1 | 1 | 0 | 6 |
| Li 2020 | 1 | | | 1 | 1 | 1 | 1 | 0 | 1 | 1 | 7 |
